# Analytical Methods for Brassinosteroid Analysis: Recent Advances and Applications

**DOI:** 10.1093/pcp/pcae038

**Published:** 2024-04-15

**Authors:** Jana Oklestkova, Miroslav Kvasnica, Miroslav Strnad

**Affiliations:** Laboratory of Growth Regulators, Faculty of Science, Palacký University & Institute of Experimental Botany, Czech Academy of Sciences, Šlechtitelů 27, Olomouc CZ-78371, Czech Republic; Laboratory of Growth Regulators, Faculty of Science, Palacký University & Institute of Experimental Botany, Czech Academy of Sciences, Šlechtitelů 27, Olomouc CZ-78371, Czech Republic; Laboratory of Growth Regulators, Faculty of Science, Palacký University & Institute of Experimental Botany, Czech Academy of Sciences, Šlechtitelů 27, Olomouc CZ-78371, Czech Republic

**Keywords:** Brassinosteroids, Chemical synthesis, Chromatography, Immunoassays, Mass spectrometry, Quantification

## Abstract

Brassinosteroids (BRs) are plant steroidal hormones that play crucial roles in plant growth and development. Accurate quantification of BRs in plant tissues is essential for understanding their biological functions. This study presents a comprehensive overview of the latest methods used for the quantification of BRs in plants. We discuss the principles, advantages and limitations of various analytical techniques, including immunoassays, gas chromatography–mass spectrometry and liquid chromatography–tandem mass spectrometry that are used for the detection and quantification of BRs from complex plant matrixes. We also explore the use of isotopically labeled internal standards to improve the accuracy and reliability of BR quantification.

## Introduction

Brassinosteroids (BRs) are a class of polyhydroxysteroids that have been recognized as a sixth class of plant hormones. They are structurally related to animal and insect steroid hormones. BRs were first explored during the 1970s when Mitchell with coworkers reported promotion in stem elongation and cell division by the treatment of organic extracts of rapeseed (*Brassica napus* L.) pollen (Mitchell et al. 1970). Brassinolide (BL) was the first BR to be isolated in 1979 (Grove et al. 1979). Since its discovery, over 70 BR compounds have been isolated from plants.

**Fig. 1:**
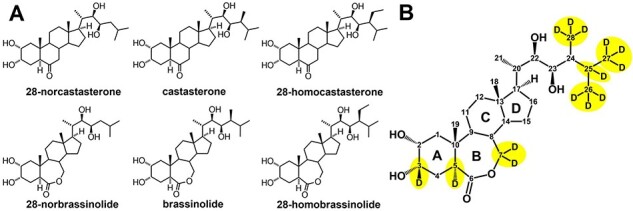
Structures of brassinosteroids. (A) Examples of natural brassinosteroids with 27, 28 and 29 carbons. (B) Structure of brassinolide with marked positions into which deuterium atoms have been introduced.

BRs have been found in all plant organs, including pollen, anthers, seeds, leaves, stems, roots, flowers and grains. They have also been detected in insect and crown galls. In general, pollen and immature seeds are particularly rich sources of BRs (1–100 ng/g fresh weight), while concentrations in vegetative tissues are very low (about 0.1 ng/g fresh weight) compared to those of other plant hormones (Bajguz and Tretyn 2003). All natural BRs are characterized by their 5α-cholestane skeleton, and their structural differences are due to the type and orientation of the oxygen functions on rings A and B (**Fig. 1A**). BRs are divided into 7-oxalactone, 6-oxo and 6-deoxo according to the arrangement on ring B. They can be further classified as C27, C28 or C29 BRs depending on the alkyl substitution at C-24 in the side chain. Furthermore, their conjugates with sugars or fatty acids have also been identified in plants (Bajguz 2011).

The structural diversity of BRs can affect their behavior in different purification and quantification methods. For instance, sometimes 6-oxo BRs show activity similar to 7-oxalactone compounds, while non-oxidized BRs exhibit essentially little activity in certain bioassays (Manghwar et al. 2022). This implies that the chemical structure of BRs can influence their detectability in bioassays, which are often used for their quantification (Kanwar et al. 2017). Moreover, the presence of conjugates and the variability in the alkyl substitution on the side chain can complicate the purification process, as these structural differences can affect the solubility and other physical and chemical properties of BRs. Therefore, the chemical structure of BRs plays a crucial role in their purification and quantification from plant material (Hou et al. 2017). If complications arise when using BR conjugates, a viable alternative is to replace them with deuterium-labeled BRs. The introduction of deuterium into the BR structure offers a significant advantage: unlike conjugates, this modification leaves the physical properties essential for liquid chromatography–mass spectrometry (LC–MS) analysis unchanged.

This review presents a detailed discussion of the analytical methods used for BR extraction, purification and quantification. It describes the principles, advantages, limitations, recent advances and applications of various techniques, including chromatographic, spectrometric and immunochemical methods.

## Immunochemical Methods

BRs are crucial for plant growth and development, but their identification and quantification in plant tissues can be a time-consuming and laborious process. Traditional chromatographic methods of the BR analysis can be lengthy and labor-intensive and require sophisticated equipment. From the historical point of view, immunoassays provide a good alternative due to the simplicity of the equipment and measuring procedure (Khripach et al. 2011). The immunochemical approach involves the use of antibodies that can specifically bind to BRs, allowing their detection and quantification (Khripach et al. 2011).

### Polyclonal antibody–based methods

Polyclonal antibodies are a heterogeneous mixture of antibodies that recognize different epitopes on the same antigen. They are produced by injecting an antigen into an animal, which then produces a series of antibodies against the antigen. Yokota et al. (1990) elucidated a robust radioimmunoassay (RIA) method for the quantification of BRs. To generate an antiserum against castasterone (CS), a rabbit was immunized with CS carboxymethyloxime conjugated to bovine serum albumin. The assay demonstrated detection limits for CS and BL of ∼0.3 pmol, exceeding the sensitivity of the rice bioassay. The challenge in achieving increased sensitivity is the increased background signal due to nonspecific binding of BRs to the test tubes. Investigation of cross-reactivity with 27 compounds, including both natural and synthetic BRs, demonstrated the specificity of the antiserum in recognizing different functional groups. Despite a notable exception where BRs with a 24-methylene group showed low cross-reactivity, it is noteworthy that the functional recognition observed in the RIA is consistent with the potent biological activities established by structure–activity relationship studies. This RIA system proved to be effective in the analysis of endogenous BRs in seeds and stems of *Phaseolus vulgaris* (Yokota et al. 1990). In addition, the antiserum derived from this study was instrumental in localizing BRs in pollen tissue by immunocytochemistry (Taylor et al. 1993). Swaczynová et al. (2007) subsequently improved the enzyme-linked immunosorbent assay (ELISA) method by using selective polyclonal antibodies against 24-epicastasterone (24-epiCS). This polyclonal antibody was able to bind to BL and 24-epibrassinolide (24-epiBL), but did not react with other plant sterols. The ELISA method was used to determine BRs in the tissues of *B. napus* and *Arabidopsis thaliana*. In addition, good agreement was found between the results obtained by the ELISA method and the high performance liquid chromatography-mass spectrometry (HPLC–MS) approach developed simultaneously (Swaczynová et al. 2007).

In recent years, three ELISA tests based on polyclonal antibodies have been developed. First, an immunochemical system for the determination of 28-homobrassinolide and 28-homocastasterone was showed by Khripach et al. (2008a). In the second case, the antibodies of the test system specifically bound to steroids containing 2α,3α-diol, 22*R*,23*R*-diol and a 7-membered 7-oxalactone B-ring (B-lactone-BR), but they had low (2–8%) cross-reactivity with the BR 6-keto series (Khripach et al. 2008b). Therefore, Pradko et al. (2015) developed a system specific for the quantification of 6-keto BRs using the conjugate of 28-norcastasterone with bovine serum albumin or horse radish peroxidase.

### Monoclonal antibody–based methods

In contrast, monoclonal antibodies are a homogeneous population of identical antibodies that recognize a single epitope on an antigen. They are produced by fusing a single B cell with a cancerous myeloma cell to create a hybridoma cell line that produces identical antibodies. Horgen et al. (1984) produced monoclonal antibodies directed against a synthetic analog of BL non-covalently linked to a carrier protein. Hybridoma clones were derived from CAF1 mice, and the resulting antibodies were used in an ELISA to study the distribution of BL in *B. napus* tissues. Despite the successful generation of antibodies, they showed considerable cross-reactivity (35–48%) with prominent plant sterols such as sitosterol, ergosterol and stigmasterol. This substantial cross-reactivity rendered the antibodies unsuitable for the analysis of BRs due to the lack of specificity for the target compounds. Anti-BR antibodies can also be used in immunoaffinity chromatography (IAC) for rapid and specific purification of BRs before the analytical methods. Using a specific monoclonal antibody prepared against BR, Oklestkova et al. (2017) developed an immunoaffinity extraction to rapidly and selectively isolate endogenous BRs containing 2α,3α-diol within the steroid molecule’s ring A. Immunoaffinity chromatography was shown to significantly reduce the matrix effect, thereby increasing the selectivity and sensitivity of subsequent ultra high performance liquid chromatography (UHPLC)–MS/MS analysis. The combination of IAC and UHPLC–MS/MS is used to detect endogenous BRs in tissue extracts of *Zea mays*, *A. thaliana, Humulus lupulus* and *B. napus* (Oklestkova et al. 2017). This method has also been used to elucidate the effect of BRs on root growth and development (Vukašinović et al. 2021), where it was found that high levels of BRs have an effect on root cell elongation, whereas lower levels promote cell division. Therefore, changes in the levels of these hormones along the root are probably used to tell the cells whether they should still divide or start to elongate.

## Chromatographic and Mass Spectrometric Methods

### Isotopically (deuterium) labeled internal standards of BRs

The use of deuterated BRs as internal standards represents an important approach that enables the precision of quantitative measurements in plant hormone research. This approach not only guarantees the reproducibility and reliability of quantitative assessments but also ensures an accurate representation of BR concentration levels in complex biological samples. Furthermore, the incorporation of deuterium into the steroidal framework provides numerous advantages over derivatization. The primary benefit lies in maintaining the same values of key physical properties with natural BRs, such as stability, polarity, solubility and retention time. Considering the significance of deuterated BRs for the HPLC–MS analysis, numerous strategies for their synthesis have been developed (Patil et al. 2015) to introduce deuterium atom(s) to several positions of the BR skeleton (**Fig. 1B**).

Due to the importance of deuterium in increasing the efficiency of the LC–MS analysis of BRs, derivatives containing three or more deuterium atoms were deliberately synthesized. This enrichment with a higher number of deuterium atoms serves to optimize the analytical accuracy of identification and quantification of BRs using the LC–MS analysis. The chemical synthesis of deuterated BRs involved multi-step reactions. A successful approach to introducing three or more deuterium atoms into the structure of a BR involved the construction of the entire side chain. This strategic method uses low–molecular weight substances containing deuterium atoms in the methyl group(s). An alternative way is sequential reduction of the carboxy group to the methyl group with lithium aluminum deuteride. This is thereby increasing the overall efficiency of the synthesis process and the final content of deuterium atoms in the structure. This strategic approach facilitated the synthesis of derivatives featuring three, six, or even up to seven deuterium atoms exclusively positioned at the C-25 (one atom), C-26 (three atoms), C-27 (three atoms) and C-28 (three atoms) sites of the side chain (Takatsuto and Ikekawa 1986a, 1986b, 1986c, Khripach et al. 2002a, 2002b, 2012a, 2012b, Antonchick et al. 2004). While the synthetic preparation of the BR derivatives with more than seven deuterium atoms is theoretically feasible, such compounds have not been synthesized to date.

When incorporating deuterium onto steroid rings, the presence of functional groups, particularly oxygen-based ones, becomes essential. For the B-ring, a straightforward exchange of hydrogens for deuterium on the alpha carbons adjacent to the 6-oxo group is achievable, catalyzed by both acids and bases. This method enables the preparation of the derivatives with one deuterium in the C-5 position and two in the C-7 position (Allevi et al. 1988, Kolbe et al. 1992). The maximum number of deuterium atoms introduced into the BR structure by such an exchange was seven. Kolbe and his coworkers thus prepared [2,2,4,4,5,7,7-^2^H_7_]-3-dehydro-24-epiteasterone with deuterium atoms on both A and B rings (Kolbe et al. 1998). However, it turned out that the resulting product had only 20% isotopic purity and contained other less deuterated analogs. The main drawback in using such derivatives lies in the potential re-exchange of deuterium for hydrogen during the chromatographic analysis, particularly when the mobile phase involves acids or bases. To address this concern, oxidizing the deuterated ketones to lactones effectively resolves the issue, especially in the context of two deuterium atoms positioned at the C-7 site. This modification significantly diminishes the likelihood of their exchange with hydrogen. An alternative procedure was employed for the synthesis of 24-epibrassinolide, ensuring the incorporation of two deuterium atoms exclusively at the C-7 position (Khripach et al. 2007). In contrast to the relative ease of introducing deuterium onto the B-ring, deuteration reactions in the case of the A-ring are less common. Marek and coworkers published a stereoselective reduction of chlorocarbonates for the specific deuteration of the 3β-position (Marek et al. 2012, 2015). Through this method, they successfully synthesized deuterated forms of both 24-epicastasterone and 24-epibrassinolide. However, it is worth noting that the presence of just one deuterium atom, as aforementioned, may not be optimal for the chromatographic analysis.

The choice of the deuteration site depends only on the complexity of the synthesis and the possibility of introducing more deuterium atoms. If the deuteration site is not affected by an undesired reaction during the chromatographic analysis (exchange of hydrogen for deuterium in addition to the carbonyl group), the choice of deuterium position does not affect the analysis.

It should be emphasized that the aforementioned deuteration methods and the preparation of BRs with deuterium incorporated into the skeleton are particularly suitable for LC–MS analyses. In the case of gas chromatography–mass spectrometry (GC–MS) analyses, derivatization of hydroxyl groups is required to increase the volatility. In this case, deuteration can be achieved using synthesized or commercially available deuterium-labeled derivatization reagents (see later).

### Derivatization-based LC–MS methods

Liquid chromatography–mass spectrometry (LC–MS) is becoming increasingly popular as a valuable technique for BR analysis. Its advantages include increased analytical sensitivity, improved selectivity and the ability to quantitatively detect multiple analytes in a single run.

Given the complexity and diversity of BRs, LC–MS can effectively separate these compounds and provide detailed information about their structure and quantity. Increasing the sensitivity and selectivity of detection can also be influenced by the choice of an appropriate extraction, purification or derivatization protocol (Liu et al. 2022). The following overview of LC–MS methods requiring derivatization is further subdivided according to the extraction/purification protocols chosen.

#### Two-dimensional solid-phase extraction.

Five BRs with high biological activity—24-epibrassinolide (24-epiBL), 24epiCS, 6-deoxo-24-epicastasterone, teasterone and typhasterol (TY)—were selected as target analytes for the analysis using online two-dimensional solid-phase extraction (2D µSPE) on column derivatization (OCD) coupled with HPLC–MS/MS (Wu et al. 2013). C18 sorbent was first combined with phenyl boronic acid sorbent to form a 2DμSPE column, thereby increasing the extraction selectivity. Then, after BRs were enriched on C18 sorbent, OCD was carried out with *m*-aminophenylboronic acid as the derivatization reagent increasing MS/MS sensitivity. This online method showed high selectivity (matrix effect, 80–124%), good repeatability (<10%), low limits of detection (LOD) (about 1 pg) and short analysis time (30 min) (Wu et al. 2013).

#### Polymer monolith microextraction.

In 2020, Wang and coworkers presented an online method for quantifying endogenous BR in plant tissues. This method involved the integration of polymer monolith microextraction, in situ derivatization and liquid chromatography–mass spectrometry (LC–MS) analysis. They modified an anionic hydrophilic interaction liquid chromatography monolithic column with 2-methyl-4-phenylaminomethylphenylboronic acid (2-methyl-4-PAMBA) through ion exchange interactions. They used this modified column as a boronate affinity medium to selectively capture, purify and derivatize BRs in a crude plant extract. The derivatization process involved the reaction of the boronic acid group of 2-methyl-4-PAMBA with the *cis*-diol group of BR. The BR derivatives were easily separated by breaking the ion exchange interactions using an acidic elution solvent. The entire process, including extraction and LC–MS analysis, was automated and completed within 50 min. The limits of detection ranged from 0.10 to 1.29 pg/ml. Finally, the proposed method was used for quantification of endogenous BL, CS, TY, 28-norbrassinolide (28-norBL) and 28-homobrassinolide in milligram plant samples including *Oryza sativa* L., *P. vulgaris* L., *Vigna unguiculata* and *A. thaliana* flowers (Wang et al. 2020).

#### Magnetic particle–based extraction.

Magnetic solid-phase extraction (MSPE) is one option for BRs extraction based on magnetic or magnetizable nanoparticles as sorbents (Wierucka and Biziuk 2014, Ding et al. 2016). Due to their magnetic properties, the sorbents easily clump together and disperse within the sample solution when an external magnetic field is applied/removed. This process simplifies the separation between the solution and the sorbents. Ding et al. (2014) created a method that integrates BR magnetic extraction and in situ derivatization together (MSPE-ISD). BRs were first extracted onto TiO_2_-coated magnetic hollow mesoporous silica sphere through hydrophilic interaction. The BR-adsorbed TiO_2_-coated magnetic hollow mesoporous silica sphere was then employed as a ‘microreactor’ for the derivatization of BRs with 4-(*N*,*N*-dimethylamino)phenylboronic acid (DMAPBA). This reduced the sample pretreatment process to 10 min. The BR derivatives are released from the magnetic sorbents using water and then analyzed by UPLC–electrospray ionization tandem mass spectrometry (ESI–MS/MS) (Ding et al. 2014).

In 2016, BRs were extracted from plant samples using mQuEChERS and polymer (4-vinylphenylboronic acid-*co*-ethylene glycol dimethacrylate)-coated Fe_3_O_4_@SiO_2_ as sequential MSPE sorbents (Ding et al. 2016). After coating the plant sample on p(4-VPBA-*co*-EGDMA)—Fe_3_O_4_@SiO_2_, 4-(*N*,*N*-dimethyamino)phenylboronic acid (4-DMAPBA) solution was employed for the in situ desorption/derivatization of BRs through a transesterification reaction, and finally, the resulting solution was subjected to LC–MS/MS for BR quantification. BRs (BL, CS, TY, 28-norCS and 28-norBL) were quantified only in a single flower or flower bud of *B. napus* L. using the proposed method (Ding et al. 2016).

Another possibility of using MSPE is the method based on boronate affinity magnetic nanoparticles, which allows direct fishing of BRs from plant matrixes (Xin et al. 2018). A boronate affinity magnetic nanoparticle solid phase extraction (SPE) method combined with UPLC–MS/MS was used to determine BRs in rice and *Arabidopsis* model plants. The levels of BL, CS, TY and 6-deoxo-CS in WS-2 wild-type and BRi1-5 mutants of *A. thaliana*, as well as the CS content ratio of different organs of the rice mutant M107 to its wild-type Nipponbare, were compared (Xin et al. 2018).

#### Boronate affinity solid-phase extraction.

Yu et al. (2018) developed a highly sensitive and selective strategy for BR profiling by stable isotope labeling liquid chromatography and multiple reaction monitoring scan mass spectrometry (SIL-LC–MRM-MS) analysis. They used a pair of stable isotope labeling agents, 4-phenylaminomethyl-benzeneboronic acid (4-PAMBA) and [^2^H_5_]-4-phenylaminomethyl-benzeneboronic acids (4-PAMBA-^2^H_5_), which reacted with C22-,C23-*cis*-diol on a BR molecule. The BRs subsequently generated two characteristic collision-induced dissociation (CID) neutral losses, which are used to establish multiple reaction monitoring (MRM)–based detection and screening. Using this SIL-LC–MRM-MS strategy, 13 BRs were successfully quantified in different tissues of rape flower (Yu et al. 2018).

Xin et al. (2013a) developed a rapid two-step SPE extraction based on mixed-mode anion exchange and cation exchange cartridges, which combine ion exchange and reversed-phase mechanisms. To enhance the MS response, the BRs were subsequently derivatized with 3-(dimethylamino)-phenylboronic acid (DMAPBA). As a result, a hybrid strategy combining UPLC–MRM^3^-MS analysis with isotope dilution-based quantification was used to profile BL, CS, teasterone and TY in 1 g of samples of rice and *Arabidopsis* and to describe the distribution of BRs in different tissues of these model plants (Xin et al. 2013a). To optimize and reduce the matrix effect, an additional step was added to the purification protocol. Boronate affinity magnetic nanoparticles (BA-MNPs) were synthesized, which specifically recognize and adsorb neutral interferents (mainly saccharides) via their affinity for boranate (Xin et al. 2013b). Although the addition of the BA-MNP treatment to the original protocol (Xin et al. 2013a) extended the overall purification time by ∼2 h, the sensitivity of the method was significantly increased (e.g. 5-fold for 24-epiBL, Xin et al. 2013b). Three years later, the method was further improved by performing BR screening on UPLC–QTrap MS and UPLC–QTof MS systems. This enabled the identification of a completely new BR precursor—6-deoxo-28-homotyphasterol—in rice panicles (Xin et al. 2016).

The method, based on pipette tip solid-phase followed by the use of a quaternary ammonium derivatization reagent, enabled a significant reduction of the amount of plant material to submilligram levels (Deng et al. 2016). The authors adopted the PT-SPE purification procedure with C18 sorbent BR isolation and purification. Subsequently, they used 4-boronic-*N*,*N*,*N*-trimethylbenzenammonium iodide (BTBA) as a chemical derivatization agent, which led to an increase in the ionization efficiency of BRs and thus a significant enhancement of the MS response. Thanks to this method (PT-SPE-UPLC–MS/MS), it was possible to quantify 24-epiBL in only 0.5 mg of FW rice leaves (Deng et al. 2016).

Other use of quaternary ammonium boronates was described by Luo et al. (2018). They used a hybrid approach that combines LC–MS with an integrated sample pretreatment method based on strong cation exchange and boronate affinity. First, the plant extract was subjected to solid-phase boronate affinity labeling. This was followed by desorption and salt phase transition extraction (SPTE) for further purification. The integration of labeling and extraction, desorption and SPTE significantly increased the rapidity, sensitivity and selectivity of this method. The LOD for six BRs ranged from 1.4 to 2.8 pg/ml (Luo et al. 2018). However, in *Arabidopsis* shoots, the authors were only able to detect one BR, 6-deoxocastasterone, which is weaker, e.g. compared to the method described by Yu et al. (2019) (**Table 1**). They synthesized a new derivatizing agent called 2-methyl-4-phenylaminomethylphenylboronic acid (2-methyl-4-PAMBA). The 2-methyl-4-PAMBA derivatives with BR showed significantly better stability in the aqueous solvent environment, especially for the C27-BR derivatives (28-norCS and 28-norBL). A subsequent method based on LC–MRM-MS and assisted labeling of 2-methyl-4-PAMBA and 2-methyl-4-PAMBA-[2H5] allowed the detection of endogenous C27-, C28- and C29-BRs in milligram plant samples, e.g. also in *Arabidopsis* shoots (Yu et al. 2019, **Table 1**).

**Table 1:** Endogenous BR concentrations (pg/g FW) obtained in *Arabidopsis* shoots or seeds by various chromatographic and mass spectrometric methods

BRs content (pg/g FW)					
		LC–MS			GC–MS
	Oklestkova et al. (2017)	Li et al. (2019)	Yu et al. (2019)	Luo et al. (2018)	Fujioka et al. (1998)
BL	40 ± 20	n.d.	4.34 ± 0.54	n.d.	1,860
28-norBL	50 ± 20	n.d.	n.d.	n.d.	n.d
CS	n.d.	n.d.	2.34 ± 0.18	n.d.	440
24-epiCS	n.d.	210 ± 30	n.d.	n.d.	n.d
28-norCS	n.d.	n.d.	2.20 ± 0.12	n.d.	n.d.
6-deoxoCS	n.d.	n.d.	n.d.	1,510 ± 70	1,570
TY	40 ± 20	n.d.	0.16 ± 0.07	n.d.	1,340

Abbreviations: 6-deoxoCS, 6-deoxocastasterone; 24-epiCS, 24-epicastasterone; n.d., not detected; 28-norCS, 28-norcastasterone.

It is also possible to use rhodamine B-boronic acid (RhB-BA) to derivatize BRs (An et al. 2020), and the LC–HV-p-MRM-MS method was proposed using a RhB-BA derivatization for the rapid determination of BRs in sample plants. The HV-p-MRM strategy was suggested based on the idea that the precursor ion of BR remains stable in collision cells and is less likely to fragment at high CID voltage. Meanwhile, coexisting impurities that are easily fragmented will break down into smaller ions, thus improving the signal-to-noise ratio of the analytes. The LOD ranged from 1.49 to 6.56 pg/ml (An et al. 2020).

A novel approach to the extraction of BRs described by Chen et al. (2018) is based on in situ the extraction/derivatization/desorption method coupled with UPLC–MS. First, the thiol-functionalized was linked to 4-mercaptophenylboronic acid (4-MPBA) via a disulfide bond. 4-MPBA selectively captured BRs from the plant extract by in situ derivatization to form BR-MPBA derivatives. The reducing agent dithiothreitol was then used to cleave the disulfide bond, allowing the BR-MPBA derivatives to be eluted from the sorbent and finally subjected to LC–MS analysis (Chen et al. 2018). Unfortunately, the starting weight of plant material for this type of analysis is relatively high (100 mg) compared to the other BR analyses mentioned.

#### Dispersive solid-phase extraction.

Another option for BR analysis published in 2019 is dispersive matrix solid-phase extraction (DMSPE) coupled with high-performance liquid chromatography–tandem mass spectrometry. The DMSPE method involves the following steps: solid samples are first combined with a solid sorbent (called as dispersant) in a microcentrifuge tube. The mixture is then centrifuged after the addition of an extraction solvent and detergents, which form another type of sorbent. In essence, DMSPE combines mechanical grinding of the sample and sorbent with efficient cleaning by centrifugation (Li et al. 2019). The authors described three different protocols of DMSPE for different plant matrix (for oilseed rape and for *A. thaliana*). After DMSPE, BRs were derivatized with BTBA and subsequently analyzed by HPLC–MS/MS. This method was also used to geographically profile the endogenous BRs of oilseed rape across various provinces in the Yangtze River Basin (Li et al. 2019).

### Non-derivatization LC–MS methods

Most of the methods mentioned earlier require the derivatization of BRs before their LC–MS analysis, mainly to improve their detection limits. However, it turns out that derivatization may not be the key to successfully detect BR, when the plant matrix contains a high level of interfering substances causing a huge chemical background (Tarkowská et al. 2014). Therefore, Tarkowská et al. (2016) developed an extremely sensitive LC–MS method capable of profiling up to 22 inherent BRs. The method achieved detection limits ranging from 0.05 to 40 pg and eliminated the need for derivatization. Extraction of BRs with 60% acetonitrile was followed by a two-step purification by SPE and finally the UHPLC analysis coupled with ESI–MS/MS (Tarkowská et al. 2016). The described method proved to be sufficiently sensitive for quantification of not only biologically active BRs themselves but also their biosynthetic precursors and metabolite. A sensitive method for the BR analysis with a very low detection limit (0.010−0.070 pg/ml) was described by Huo et al. (2021). They extracted BRs by online solid-phase extraction using the boronic acid–functionalized Scholl coupling microporous polymer (SMP). The polymer was synthesized by Scholl coupling and then Schiff’s reaction with 4-formylphenylboronic acid to form boronic acid–functionalized B-SMP. This B-SMP was then packed into a SPE cartridge and coupled to HPLC–MS/MS and successfully used to analyze BL, 24-epiBL and 28-homobrassinolide in 15 plant-derived foodstuffs (Huo et al. 2021).

### Gas chromatography–mass spectrometry

BRs can also be analyzed by gas chromatography, which uses an inert or unreactive gas (a carrier gas), as a mobile phase. Since BRs are not volatile compounds, they must be derivatized prior to the GC–MS analysis, for which methylboronic acid in pyridine is most often used. Derivatization can be also carried out by methyl boronation of the side chain followed by trimethylsilylation of C-3-hydroxy groups (Takatsuto 1994, Kanwar et al. 2017).

GC–MS of BRs experienced its greatest development in the 80s and 90s of the last century when, e.g. some biosynthetic precursors of BL were identified using this method (Schmidt et al. 1993, Abe et al. 1995, Takatsuto et al. 1999). In recent years, the GC–MS analysis has been used to describe the biosynthetic relationship between C28- and C29-BRs in rice (*Oryza sativa*) seedlings (Joo et al. 2015) and also helped to reveal the CS phosphorylation process, as an important conjugation process for maintaining the homeostatic level of active BRs in *A. thaliana* and *Lycopersicum esculentum* plants (Kim et al. 2015). One of the drawbacks of GC–MS is that it requires a greater amount of plant material for extractions compared to LC–MS methods. For instance, Takatsuto et al. (1989) used 2.9 kg of sunflower pollen to identify BL, CS and 28-nor CS in *Helianthus annuus* L., while Yokot and coworkers used 900 g of wheat grain to identify 3-dehydroteasterone and 3,6-diketobrassinosteroids (Yokota et al. 1994). Although it has gradually been possible to reduce the amount of plant material required to gram quantities (e.g. 10 g of *Arabidopsis* seeds, Fujioka et al. 1998, **Table 1**.), this is still incomparable to the milligram and submilligram weights of plant material needed for LC–MS analysis (**Table 2**).

**Table 2:** Overview of LC–MS methods for the analysis of BRs developed in the last 10 years

Pretreatment	Detection	LOD (pg/ml)	Amount of plant tissues (mg)	Plant tissues	Reference
Derivatization methods					
mSiO_2_-SS-PBA nanofiber	UPLC–MS	0.21–0.62	100	*Humulus lupulus, A. thaliana, O. sativa*	Chen et al. 2018
2-Methyl-4-PAMBA	LC–MRM-MS	0.1–1.68	1	*Oryza sativa, A. thaliana, B. napus*	Yu et al. 2019
BA-MNPS	UPLC–MS/MS	8.6–34.8	10	*Oryza sativa, A. thaliana*	Xin et al. 2018
RhB-CID (HV-P-MRM)	LC–MS	1.49–6.56	10	*Brassica napus*	An et al. 2020
Solid-phase boronate affinity labeling-SPTE	UPLC–ESI–MS/MS	1.4–2.8	10–20	*Oryza sativa, A. thaliana, B. napus*	Luo et al. 2018
mQuEChERS-4-DMAPBA	UPLC–MS/MS	0.27–1.29	100	*Oryza sativa, B. napus*	Ding et al. 2016
Online 2DμSPE-OCD	HPLC–MS/MS	1.4–6.6	225	*Lycopersicon esculentum*	Wu et al. 2013
4-PAMBA + d_5_4-PAMBA	SIL-LC–MRM-MS	Not specified	50	*Brassica napus*	Yu et al. 2018
Online PMME-ISD (2-methyl-4-PAMBA	LC–MS	0.1–1.29	1	*Oryza sativa, P. vulgaris*, *V. unguiculata*, *A. thaliana*	Wang et al. 2020
PT-SPE-BTBA	UPLC–MS/MS	0.013–0.042	0.5–5	*Oryza sativa*	Deng et al. 2016
DMSPE-BTBA	HPLC–MS/MS	1.38 − 6.75	0.5–2	*Arabidopsis thaliana, B. napus*	Li et al. 2019
Non-derivatization methods					
Two-step SPE	UPLC–ESI–MS/MS	0.05–10	50	*Brassica napus*	Tarkowská et al. 2016
SPE-IAC	UPLC–ESI–MS/MS	1–50	50	*Zea mays, A. thaliana, H. lupulus, P. vulgaris*	Oklestkova et al. 2017
Online-SPE	HPLC–MS/MS	0.01–0.07	1,000	Various fruits and vegetables	Huo et al. 2021

## Other Methods

In the last decade, complex quantitative proteomic analyses have been increasingly used to analyze the function of phytohormones. Two-dimensional difference gel electrophoresis coupled to mass spectrometry was used to identify BR-regulated proteins in *Arabidopsis* (Deng et al. 2007). This technique identified 42 BR-regulated proteins predicted to play a potential role in BR regulation of specific cellular processes such as signaling, cytoskeletal rearrangement, vesicle trafficking and phytohormone and vitamin biosynthesis (Deng et al. 2007). The regulatory role of BRs in rice seed germination was elucidated using the iTRAQ-based quantitative proteomic analysis (isobaric tags for relative and absolute quantification), which identified more than 800 BR-responsive proteins. These results not only significantly enriched the BR-regulated protein database in rice but also provided new insights into the regulatory role of BRs in rice seed germination (Li et al. 2016). Quantitative proteomics is an essential part of the toolbox for studying regulatory processes in plants, such as growth and development (Yan et al. 2022).

While liquid chromatography–mass spectrometry (LC–MS) remains a powerful analytical tool widely used in plant research for biomolecule profiling and quantification, it is limited by the spatial distribution of target molecules within samples. The homogenization process during sample preparation removes spatial information. Mass spectrometric imaging coupled with desorption electrospray ionization mass spectrometry (DESI) offers a solution. DESI enables label-free, multiplexed and objective measurements of molecular targets directly from complex surfaces. By analyzing samples without prior homogenization, researchers gain spatially resolved molecular insights (Wiseman et al. 2008). For example, Zhang et al. (2021) used DESI–mass spectrometric imaging to study the distribution and abundance variations of plant hormones (jasmonic acid, auxins and abscisic acid), their precursors and metabolites in wounded leaves and leaf-print TLC plates. Their results not only revealed distinct levels of phytohormones between wounded and unwounded leaves (as detected by LC–MS/MS analysis) but also highlighted heterogeneous distributions and strong correlations within wounded regions. Anticipated future applications include the extension of this method to other phytohormone groups, including BRs.

## Concluding Remarks

In summary, the BR analysis has made great strides in recent years, particularly in the area of sample pretreatment and the sensitivity and precision of the analysis itself. Sophisticated methods for BR purification and subsequent derivatization have significantly reduced the amount of plant material required (down to submicrogram levels) and the overall analysis time (summarized in **Table 2**). Technological advances in the development of chromatographic and spectrometric instrumentation for the final quantification of BRs have also allowed the detection limits, for LC–MS to be reduced to 0.01 pg/ml (Huo et al. 2021). Further advances in the BR analysis are expected to contribute to a better understanding of the role of BRs in plant development and a detailed description of the branched BR biosynthetic pathway.

## Data Availability

No new data were generated in this review article.

## References

[R1] Abe H., Takatsuto S., Nakayama M. and Yokota T. (1995) 28-Homotyphasterol, a new natural brassinosteroid from rice (*Oryza sativa* L.) *Bran*. *Biosci. Biotechnol., Biochem*. 59: 176–178.

[R2] Allevi P., Anastasia M., Cerana R., Ciuffreda P. and Lado P. (1988) 24-Epibrassinolide uptake in growing maize root segments evaluated by multiple-selected ion monitoring. *Phytochemistry* 27: 1309–1313.

[R4] An N., Zhu Q.F., Yu L., Chen Y.T., Chen S.L. and Feng Y.Q. (2020) Derivatization assisted LC-p-MRM-MS with high CID voltage for rapid analysis of brassinosteroids. *Talanta* 217: 121058. 10.1016/j.talanta.2020.121058 32498827

[R3] Antonchick A.P., Schneider B., Zhabinskii V.N. and Khripach V.A. (2004) Synthesis of [26,27-^2^H_6_]brassinosteroids from 23,24-bisnorcholenic acid methyl ester. *Steroids* 69: 617–628. 15465106 10.1016/j.steroids.2004.05.014

[R5] Bajguz A. (2011) Brassinosteroids—occurrence and chemical structures in plants. *In* Brassinosteroids: A Class of Plant Hormone. Edited by Hayat, S. and Ahmad, A. pp. 375–395. Springer, London.

[R6] Bajguz A. and Tretyn A. (2003) The chemical characteristic and distribution of brassinosteroids in plants. *Phytochemistry* 62: 1027–1046. 12591256 10.1016/s0031-9422(02)00656-8

[R7] Chen M., wang R., Zhu Y., Liu M., Zhu F. and Xiao J. (2018) 4-Mercaptophenylboronic acid-modified spirally-curved mesoporous silica nanofibers coupled with ultra performance liquid chromatography–mass spectrometry for determination of brassinosteroids in plants. *Food Chem*. 263: 51–58. 29784327 10.1016/j.foodchem.2018.04.129

[R8] Deng T., Wu D., Duan C. and Guan Y. (2016) Ultrasensitive quantification of endogenous brassinosteroids in milligram fresh plant with a quaternary ammonium derivatization reagent by pipette-tip solid-phase extraction coupled with ultra-high-performance liquid chromatography tandem mass spektrometry. *J. Chromatogr. A* 1456: 105–111. 27338695 10.1016/j.chroma.2016.06.026

[R9] Deng Z., Zhang X., Tang W., Oses-Prieto J.A., Suzuki N., Gendron J.M., et al. (2007) A proteomics study of brassinosteroid response in *Arabidopsis*. *Mol. Cell. Proteom*. 6: 2058–2071. 10.1074/mcp.M700123-MCP200 PMC2966871 17848588

[R10] Ding J., Mao L.J., Guo N., Feng Y.Q. and Feng Y.-Q. (2016) Determination of endogenous brassinosteroids using sequential magnetic solid phase extraction followed by *in situ* derivatization/desorption method coupled with liquid chromatography–tandem mass spectrometry. *J. Chromatogr. A* 1446: 103–113. 27072523 10.1016/j.chroma.2016.04.012

[R11] Ding J, Wu J, Liu J, Yuan B and Feng Y. (2014) Improved methodology for assaying brassinosteroids in plant tissues using magnetic hydrophilic material for both extraction and derivatization. *Plant Methods*. 10: 39 10.1186/1746-4811-10-39 PMC4350586 25745510

[R12] Fujioka S., Noguchi T., Yokota T., Takatsuto S. and Yoshil S. (1998) Brassinosteroids in *Arabidopsis thaliana*. *Phytochemistry* 48: 595–599. 9664702 10.1016/s0031-9422(98)00065-x

[R13] Grove M.D., Spencer G.F., Rohwedder W.K., Mandava N., Worley J.F., Warthen J.D. Jr., et al. (1979) Brassinolide, a plant growth-promoting steroid isolated from *Brassica Napus* pollen. *Nature* 281: 216–217.

[R14] Horgen P.A., Nakagawa C.H. and Irvin R.T. (1984) Production of monoclonal antibodies to a steroid plant growth regulator. *Can. J. Biochem. Cell Biol* 62: 715–721.

[R15] Hou Y., Qiu J., Wang Y., Li Z., Zhao J., Tong X., et al. (2017) A quantitative proteomic analysis of brassinosteroid-induced protein phosphorylation in rice (*Oryza sativa* L.). *Front. Plant Sci*. 8: 514. 10.3389/fpls.2017.00514 PMC5383725 28439285

[R16] Huo S., Song X., Li L., Wang R., Wang X. and Ji W. (2021) Boronic acid-functionalized scholl-coupling mesoporous polymers for online solid-phase extraction of brassinosteroids from plant-derived foodstuffs. *J. Agric. Food Chem*. 69: 4883–4893. 33847497 10.1021/acs.jafc.1c00211

[R17] Joo S.H., Jang M.S., Kim M.K., Lee J.E. and Kim S.K. (2015) Biosynthetic relationship between C_28_-brassinosteroids and C_29_-brassinosteroids in rice (*Oryza sativa*) seedlings. *Phytochemistry* 111: 84–90. 25433632 10.1016/j.phytochem.2014.11.006

[R18] Kanwar M.K., Bajguz A., Zhou J. and Bhardway R. (2017) Analysis of brassonosteroids in plants. *J. Plant Growth Regul*. 36: 1002–1030.

[R19] Khripach V.A., Khripach N.B., Zhabinskii V.N., Zhiburtovich Y.Y., Schneider B. and De Groot A. (2007) Synthesis of [7,7-^2^H_2_]epibrassinolide. *J. Label. Compd. Radiopharm*. 50: 1153–1158.

[R20] Khripach V.A., Litvinovskaya R.P., Raiman M.E., Drach S.V., Zhabinskii V.N., Sviridov O.V., et al. (2008a) Synthesis and immunochemical determination of 28-homobrassinosteroids. *Vesti NAN Belarusi, ser khim navuk*. 1: 47–58.

[R21] Khripach V.A., Zhabinskii V.N., Antonchick A.P., Konstantinova O.V. and Schneider B. (2002b) Synthesis of hexadeuterated 23-dehydroxybrassinosteroids. *Steroids* 67: 1101–1108. 12441196 10.1016/s0039-128x(02)00071-5

[R22] Khripach V., Zhabinskii V., Antonchick A., Litvinovskaya R., Drach S., Sviridov O., et al. (2008b) A new type of modified brassinosteroids for enzyme-linked immunosorbent assay. *Nat. Prod. Commun*. 3: 735–748.

[R23] Khripach V.A., Zhabinskii V.N., Ermolovich Y.V. and Gulyakevich O.V. (2012b) Synthesis of [26-^2^H_3_]-campesterin and [26-^2^H_3_]-campestanol, deuterated analogs of biosynthetic precursors of 28C-brassinosteroids. *Chem. Nat. Compd*. 48: 606–609.

[R24] Khripach V.A., Zhabinskii V.N., Gulyakevich O.V. and Ermolovich Y.V. (2012a) Synthesis of [26-^2^H_3_]-6-deoxo-24-epicastasterone. *Chem. Nat. Compd*. 48: 601–605.

[R25] Khripach V.A., Zhabinskii V.N., Konstantinova O.V., Khripach N.B., Antonchick A.P. and Schneider B. (2002a) Synthesis of [26-^2^H_3_]brassinosteroids. *Steroids* 67: 587–595. 11996931 10.1016/s0039-128x(02)00004-1

[R26] Khripach V.A. Zhabinskii V.N. Litvinovskaya R.P. (2011) Immunoassay of brassinosteroids. *In* Brassinosteroids: A Class of Plant Hormone. Edited by Hayat, S. and Ahmad, A. pp. 375–395. Springer, London.

[R27] Kim M.K., Jang M.S., Youn J.H., Son S.H., Lee J.E., Kim T.W., et al. (2015) Occurrence of phosphorylated castasterone in *Arabidopsis thaliana* and *Lycopersicum esculentum*. *Physiol. Plant*. 153: 58–67. 24939035 10.1111/ppl.12242

[R28] Kolbe A., Marquardt V. and Adam G. (1992) Synthesis of tritium labelled 24-epibrassinolide. *J. Label. Compd. Radiopharm*. 31: 801–805.

[R29] Kolbe A., Schneider B., Voigt B. and Adam G. (1998) Labelling of biogenetic brassinosteroid precursors. *J. Label. Compd. Radiopharm*. 41: 131.

[R30] Li Y., Deng T., Duan C., Ni L., Wang N. and Guan Y. (2019) Dispersive matrix solid-phase extraction method coupled with high performance liquid chromatography-tandem mass spectrometry for ultrasensitive quantification of endogenous brassinosteroids in minute plants and its application for geographical distribution study. *J. Agric. Food Chem*. 67: 3037–3045. 30821966 10.1021/acs.jafc.8b07224

[R31] Liu X., Zhong Y., Li W., Li G., Jin N., Zhao X., et al. (2022) Development and comprehensive SPE-UHPLC-MS/MS analysis optimization, comparison, and evaluation of 2,4-epibrassinolide in different plant tissues. *Molecules* 27: 831. 10.3390/molecules27030831 PMC8839131 35164096

[R32] Li Q.F., Xiong M., Xu P., Huang L.C.H., Zhang C.Q., Liu Q.-Q., et al. (2016) Dissection of brassinosteroid-regulated proteins in rice embryos during germination by quantitative proteomics. *Sci. Rep*. 6: 34583. 10.1038/srep34583 PMC5050409 27703189

[R33] Luo X.T., Cai B.D., Yu L., Ding J. and Feng Y.Q. (2018) Sensitive determination of brassinosteroids by solid phase boronate affinity labeling coupled with liquid chromatography-tandem mass spektrometry. *J. Chromatogr. A* 1546: 10–17. 29525124 10.1016/j.chroma.2018.02.058

[R34] Manghwar H., Hussain A., Ali Q. and Liu F. (2022) Brassinosteroids (BRs) role in plant development and coping with different stresses. *Int. J. Mol. Sci*. 23: 1012. 10.3390/ijms23031012 PMC8835148 35162936

[R35] Marek A., Klepetarova B. and Elbert T. (2015) A facile method for steroid labeling by heavy isotopes of hydrogen. *Tetrahedron* 71: 4874–4882.

[R36] Marek A., Patil M.R., Klepetarova B., Kohout L. and Elbert T. (2012) A stereospecific pathway for the introduction of deuterium on the brassinosteroid skeleton by reductive dechlorination of chlorocarbonates. *Tetrahedron Lett*. 53: 2048–2050.

[R37] Mitchell J.W., Mandava N.B., Worley J.F., Plimmer J.R. and Smith M.V. (1970) Brassins: a new family of plant hormones from rape pollen. *Nature* 225: 1065–1066. 16056912 10.1038/2251065a0

[R38] Oklestkova J., Tarkowská D., Eyer L., Elbert T., Marek A., Smržová Z., et al. (2017) Immunoaffinity chromatography combined with tandem mass spectrometry: a new tool for the selective capture and analysis of brassinosteroid plant hormones. *Talanta* 170: 432–440. 28501193 10.1016/j.talanta.2017.04.044

[R39] Patil M.R., Elbert T. and Keri R.S. (2015) Labelling of brassinosteroids by isotopes of hydrogen and carbon. *RSC Adv*. 5: 39726–39745.

[R40] Pradko A.G., Litvinovskaya R.P., Sauchuck A.L., Drach S.V., Baranovsky A.V., Zhabinskii V.N., et al. (2015) A new ELISA for quantification of brassinosteroids in plants. *Steroids* 97: 78–86. 25201263 10.1016/j.steroids.2014.08.022

[R41] Schmidt J., Yokota T., Spengler B. and Adam G. (1993) 28-Homoteasterone, a naturally occurring brassinosteroid from seeds of *Raphanus sativus*. *Phytochemistry* 34: 391–392.

[R42] Swaczynová J., Novák O., Hauserová E., Fuksová K., Šíša M., Kohout L., et al. (2007) New techniques for the estimation of naturally occurring brassinosteroids. *J. Plant Growth Regul*. 26: 1–14.

[R43] Takatsuto S. (1994) Brassinosteroids: distribution in plants, bioassays, and micro-analysis by gas chromatography-mass spectrometry. *J. Chromatogr. A* 658: 3–15.

[R44] Takatsuto S. and Ikekawa N. (1986a) Synthesis of [26, 28-^2^H_6_]brassinolide, [26, 28-^2^H_6_]castasterone, [26, 28-^2^H_6_]typhasterol, and [26, 28-^2^H_6_]teasterone. *Chem. Pharm. Bull*. 34: 1415–1418.

[R45] Takatsuto S. and Ikekawa N. (1986b) Synthesis of deuterio-labelled brassinosteroids, [26, 28-^2^H_6_]brassinolide, [26, 28-^2^H_6_]castasterone, [26, 28-^2^H_6_]typhasterol, and [26, 28-^2^H_6_]teasterone. *Chem. Pharm. Bull*. 34: 4045–4049.

[R46] Takatsuto S. and Ikekawa N. (1986c) Synthesis of [26,28-^2^H_6_]crinosterol, a synthetic intermediate of [26,28-^2^H_6_]brassinolide and [26,28-^2^H_6_]castasterone. *J. Chem. Soc., Perkin Trans*. 1: 591–593.

[R47] Takatsuto S., Kosuga N., Abe B., Noguchi T., Fujioka S. and Yokota T. (1999) Occurrence of potential brassinosteroid precursor steroids in seeds of wheat and foxtail millet. *J. Plant Res*. 112: 27–33.

[R48] Takatsuto S., Yokota T., Omote K., Gamoh K. and Takahashi N. (1989) Identification of brassinolide, castasterone and n orcastasterone (brassinone) in sunflower (*Helianthus annuus* L.) pollen. *Agric. Biol. Chem*. 53: 2177–2180.

[R49] Tarkowská D., Novák O., Floková K., Tarkowski P., Turečková V., Grúz J., et al. (2014) Quo vadis plant hormone analysis? *Planta* 240: 55–76. 24677098 10.1007/s00425-014-2063-9

[R50] Tarkowská D., Novák O., Oklestkova J. and Strnad M. (2016) The determination of 22 natural brassinosteroids in a minute sample of plant tissue by UHPLC–ESI–MS/MS. *Anal. Bioanal. Chem*. 408: 6799–6812. 27531032 10.1007/s00216-016-9807-2

[R51] Taylor P., Spuck K., Smith P., Sasse J., Yokota T., and Griffiths P., et al. (1993) Detection of brassinosteroids in pollen of Lolium perenne L. by immunocytochemistry *Planta* 189: 91–100

[R52] Vukašinović N., Wang Y., Vanhoutte I., Fendrych M., Guo B., Kvasnica M., et al. (2021) Local brassinosteroid biosynthesis enables optimal root growth. *Nat. Plants* 7: 619–632. 34007032 10.1038/s41477-021-00917-x

[R53] Wang X.Y., Xiong C.F., Ye T.T., Ding J. and Feng Y.O. (2020) Online polymer monolith microextraction with in-situ derivatization for sensitive detection of endogenous brassinosteroids by LC-MS. *Microchem. J*. 158: 105061.

[R54] Wierucka M. and Biziuk M. (2014) Application of magnetic nanoparticles for magnetic solid-phase extraction in preparing biological, environmental and food samples. *TrAC-Trends Anal. Chem*. 59: 50–58.

[R55] Wiseman J., Ifa D., Venter A. and Cooks R.G. (2008) Ambient molecular imaging by desorption electrospray ionization mass spectrometry. *Nat. Protoc*. 3: 517–524. 18323820 10.1038/nprot.2008.11

[R56] Wu Q., Wu D., Shen Z., Duan C. and Guan Y. (2013) Quantification of endogenous brassinosteroids in plant by on-line two-dimensional microscale solid phase extraction-on column derivatization coupled with high performance liquid chromatography–tandem mass spectrometry. *J. Chromatogr. A* 1297: 56–63. 23702098 10.1016/j.chroma.2013.04.043

[R57] Xin P., Li B., Yan J. and Ch Y. (2018) Pursuing extreme sensitivity for determination of endogenous brassinosteroids through direct fishing from plant matrices and eliminating most interferences with boronate affinity magnetic nanoparticles. *Anal. Bioanal. Chem*. 410: 1363–1374. 29238862 10.1007/s00216-017-0777-9

[R58] Xin P., Yan J., Fan J., Chu J. and Yan C. (2013a) An improved simplified high-sensitivity quantification method for determining brassinosteroids in different tissues of rice and *Arabidopsis*. *Plant Physiol*. 162: 2056–2066. 23800992 10.1104/pp.113.221952 PMC3729782

[R59] Xin P., Yan J., Fan J., Chu J. and Yan C. (2013b) A dual role of boronate affinity in high-sensitivity detection of vicinal diol brassinosteroids from sub-gram plant tissues via UPLC-MS/MS. *Analyst* 138: 1342–1345. 23340859 10.1039/c3an36533f

[R60] Xin P., Yan J., Li B., Fang S., Fan J., Tian H., et al. (2016) A comprehensive and effective mass spectrometry-based screening strategy for discovery and identification of new brassinosteroids from rice tissues. *Front. Plant Sci*. 7: 1786. 10.3389/fpls.2016.01786 PMC5127834 27965691

[R61] Yan S., Bhawal R., Yin Z., Thannhauset T.W. and Zhang S. (2022) Recent advances in proteomics and metabolomics in plants. *Mol. hortic*. 2: 17. 10.1186/s43897-022-00038-9 PMC10514990 37789425

[R62] Yokota T., Nakayama M., Wakisaka T., Schmidt J. and Adam G. (1994) 3-Dehydroteasterone, a 3,6-diketobrassinosteroid as a possible biosynthetic intermediate of brassinolide from wheat grain. *Biosci. Biotechnol. Biochem*. 58: 1183–1185.

[R63] Yokota T., Watanabe S., Ogino Y., Yamaguchi I. and Takahashi N. (1990) Radioimmunoasay for brassinosteroids and its use for comparative analysis of brassinosteroids in stems and seeds of *Phaseolus vulgaris*. *J. Plant Growth Regul*. 9: 151–159.

[R64] Yu L., Cai W.J., Ye T. and Feng Y.Q. (2019) A new boronic acid reagent for the simultaneous determination of C_27_-, C_28_-, and C_29_-brassinosteroids in plant tissues by chemical labeling-assisted liquid chromatography-mass spectrometry. *Anal. Bioanal. Chem*. 411: 1623–1632. 30715574 10.1007/s00216-019-01612-9

[R65] Yu L., Ye T., Bai Y.L., Cai W.J., Ding J., Yaun B.F., et al. (2018) Profiling of potential brassinosteroids in different tissues of rape flower by stable isotope labeling – liquid chromatography/mass spectrometry analysis. *Anal. Chim. Acta* 1037: 55–62. 30292315 10.1016/j.aca.2017.08.038

[R66] Zhang C., Žukauskaitė A., Petřík I., Pěnčík A., Hönig M., Grúz J., et al. (2021) *In situ* characterisation of phytohormones from wounded *Arabidopsis* leaves using desorption electrospray ionisation mass spectrometry imaging. *Analyst* 146: 2653–2663. 33661255 10.1039/d0an02118k

